# ICalled-DIY Device for Hands-On and Low-Cost Adapted Emergency Call Learning: A Simulation Study

**DOI:** 10.3390/children12030282

**Published:** 2025-02-26

**Authors:** Luis Castro-Alonso, Sheila Vázquez-Álvarez, Santiago Martínez-Isasi, María Fernández-Méndez, Luz Rey-Fernández, María García-Martínez, Adriana Seijas-Vijande, Roberto Barcala-Furelos, Martín Otero-Agra

**Affiliations:** 1 Complexo Hospitalario of Pontevedra, Sergas, 36002 Pontevedra, Spain; 2 REMOSS Research Group, Faculty of Education and Sport Sciences, University of Vigo, 36005 Pontevedra, Spainroberto.barcala@uvigo.gal (R.B.-F.);; 3 School of Nursing of Pontevedra, University of Vigo, 36001 Pontevedra, Spain; 4 CLINURSID Research Group, University of Santiago de Compostela, 15782 Santiago de Compostela, Spain; 5 SICRUS Research Group, Santiago de Compostela Health Research Institute (IDIS), University School of Nursing of Santiago de Compostela, 15706 Santiago de Compostela, Spain; 6 Faculty of Nursing, University of Santiago de Compostela, 15782 Santiago de Compostela, Spain; 7 Consellería de Educación, Ciencia, Universidades de Formación Profesional, Xunta de Galicia, 15781 Pontevedra, Spain

**Keywords:** emergency call, school, BLS, didactical tool, low-cost, simulation, curriculum

## Abstract

**Objective**: To assess the efficacy of a low-cost, Do-It-Yourself training material for emergency call simulation training, compared to a more traditional approach. **Methods**: A quasi-experimental design without pre-test was used. A final sample of 762 schoolchildren, aged three to twelve years, received two training programmes. The control group (C-G) received training using an adult dummy and an authentic smartphone (336 schoolchildren). The experimental group (ICall-G) was trained using a stuffed toy and the ICalled-DIY device, a low-cost simulation consisting of three sheets of paper held together by a ring that simulates a smartphone. The 20 min training was delivered by a nurse using a didactic–demonstration–simulation methodology. The evaluation consisted of a simulation scenario, in which participants had to identify the emergency and make a call and were then evaluated with a checklist. **Results**: No statistically significant differences were observed between the two groups in unlocking the phone (ICall-G: 84% vs. C-G: 83%; *p* = 0.78) or dialling 112 to make the call (ICall-G: 91% vs. C-G: 91%; *p* = 0.89). Hands-free activation in ICall-G was significantly higher (81%) compared to C-G (54%) (*p* < 0.001). At the pre-primary level, results were lower than those observed in primary education, with minimal differences between the first cycle of primary education and subsequent cycles. **Conclusions**: The use of a practical, low-cost and adapted tool for emergency call instruction was found to be comparable to a conventional approach. In addition, the use of the ICalled-DIY device was found to be more effective in facilitating the understanding of hands-free activation.

## 1. Introduction

A consensus has been reached among international organisations working on out-of-hospital cardiac arrest (OHCA) that first aid and basic life support (BLS) content and skills training should be reinforced at the school level [1]. Consequently, in the relatively near future, this population of trained young people could become an adult population with greater possibilities of attending emergencies, which would lead to an improvement in the survival probabilities of victims [2].

In this regard, the European Resuscitation Council (ERC) programme, KIDS SAVE LIVES, is developing an age-based curriculum for the teaching of BLS in all schools [1]. In this respect, in addition to cardiopulmonary resuscitation (CPR), there are other elements of first aid and BLS that do not require physical or psychomotor development and there is already evidence to suggest that these elements can be assimilated at younger ages than 11–12 years old [1,3,4,5]. The KIDS SAVE LIVES programme itself emphasises the importance of fostering interest in BLS, familiarising children with the emergency number, teaching them how to make a call in an emergency situation and ensuring that they are able to provide accurate information to dispatchers from an early age [1].

However, in practice, the implementation of BLS education is far from optimal. In order to address the current situation, it is essential to gain an understanding of the key topics that should be prioritised in training, the most effective and/or efficient strategies for teaching children, and the ages at which the various competencies can be assimilated. In response to this, over recent years, a variety of strategies have been developed which are tailored to the age of schoolchildren, as well as low-cost or do-it-yourself (DIY) teaching tools [6,7,8,9,10,11].

The objective of this study was to assess the efficacy of a strategy based on low-cost DIY materials for practical and adapted simulation in emergency call training, in comparison to a more traditional simulation strategy. Moreover, the efficacy of the aforementioned strategy was evaluated across the various stages of the educational continuum.

## 2. Materials and Methods

### 2.1. Design

A quasi-experimental and cross-sectional simulation study was conducted without a pretest. The research was approved by the Ethics Committee of the Faculty of Education and Sport Sciences at the University of Vigo (Spain) (code: 09-170123). The legal guardians of the participating children provided written informed consent.

### 2.2. Sample

The initial sample comprised 906 students aged between 3 and 12 years from five public schools in Galicia (Spain). Two nonrandomized intervention groups were established to conduct the study: three schools were assigned to the control group (n = 467 participants) and two schools were assigned to the experimental group (n = 439 participants).

In order to be included in the study, participants had to be enrolled at the schools where the research were conducted and attend the theoretical and practical training sessions. The exclusion criteria were failure to attend any of the scheduled training or evaluation sessions and failure to provide signed informed consent from the legal guardians of the participants.

Consequently, a total of 144 students were excluded from the study, resulting in a final sample of 762 students included in the analysis. Of these, 336 were assigned to the control group and 426 to the experimental group. For the purposes of the stage analysis, participants were classified in accordance with the prevailing distribution of cycles within the Spanish national system [12] (kindergarten cycle: KG-C/primary education first cycle: 1st-C/second cycle: 2nd-C/third cycle: 3rd-C). The classification of participants by academic cycle is presented in Table 1.

### 2.3. Intervention

**Figure 1:**
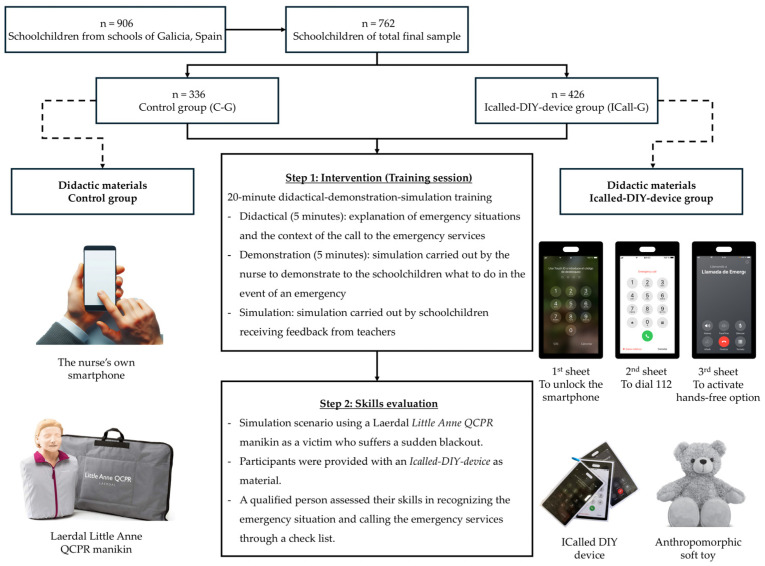
Flow chart.

The participants received training in accordance with the guidelines set forth by the ERC in 2021 [13]. The training sessions were conducted by a nurse with expertise in BLS training and in collaboration with a kindergarten/primary school teacher. This teaching team strategy was implemented to ensure similar presentations in all sessions (led by the nurse) and to have the teaching experience and knowledge of the students (provided by the student’s teacher). Each session lasted approximately 20 min (Figure 1 and Table 2). The session commenced with a theoretical explanation of the recognition of an emergency situation and the actions to be taken to alert the emergency dispatcher via a mobile phone call (5 min). This was followed by a practical demonstration of the aforementioned concepts (5 min). Afterwards, the participants engaged in a practical simulation with a victim, receiving feedback from the instructors (10 min). The maximum student–nurse ratio was 25:1. The two groups participating in the study employed different didactic tools in the course of the intervention.

The control group (C-G) used a Little Anne QCPR manikin from Laerdal (Stavanger, Norway) to simulate the victim and a genuine smartphone to perform the practical simulation of the emergency call. In this group, the nurse provided a demonstration with the manikin and simulated the call with her smartphone. Subsequently, the participants performed a simulation using the same manikin as the simulated victim and the nurse’s smartphone to practice the call. In this scenario, it was imperative to terminate the use of the smartphone prior to initiating the emergency call in order to prevent any potential errors, which necessitated the nurse’s supervision.

In the experimental group, participants were requested to attend the educational facility with their own anthropomorphic soft toy, which was to be used as a proxy for the victim. The ICalled-DIY device was employed to simulate the call to the emergency services. The experimental group was provided with a cost-effective training methodology and designated the ICalled-DIY device group (ICall-G). The ICalled-DIY device (Figure 1) is a three-sheet plastic mock-up of a smartphone with a connecting ring, which visually represents the following scenarios. The cost per unit is approximately EUR 2. The initial sheet depicts the screen of a locked smartphone, prompting the students to press the unlock button in order to initiate an emergency call. The second slide corresponds to the dialling screen for emergency calls, where participants were required to dial the emergency number (112 in Europe) and then press the button to initiate the call. The third slide depicts the call control screen, which requires the students to press the button that activates the loudspeaker mode while maintaining their hands free to follow the instructions of the emergency services. In this group, the nurse demonstrated the procedure for making an emergency call with the ICalled-DIY device using her own soft toy as an example. Subsequently, the participants engaged in a simulation in which their soft toy assumed the role of the victim and made a call with the ICalled-DIY device, while simultaneously receiving feedback from the instructor.

### 2.4. Evaluation

A skills assessment was conducted one day following the training session. This was accomplished through the use of the Little Anne QCPR manikin from Laerdal (Stavanger, Norway), the ICalled-DIY device, and a checklist of activities to be performed by the participants. The assessment comprised a simulation scenario in which the participant was required to respond to the collapse of a family member. The call to the emergency services and the subsequent dialogue with the emergency operator were evaluated.

### 2.5. Statistical Analysis

All analyses were conducted using IBM SPSS Statistics version 21 software for Windows (Armonk, NY, USA). The variables were described using both absolute and relative frequencies. For comparisons between groups, the Chi-square test was employed, with a significance level of *p* = 0.05. For comparisons between different training cycles, the Chi-square test with Bonferroni correction was employed, with a value of *p* = 0.0083. For all comparisons, effect sizes (ES) and the 95% confidence interval were calculated in accordance with Cramer’s V test: (0.10–0.30) small effect size; (0.30–0.50) moderate effect size; (≥0.50) large effect size.

## 3. Results

### 3.1. Exploratory Knowledge Analysis

**Figure 2:**
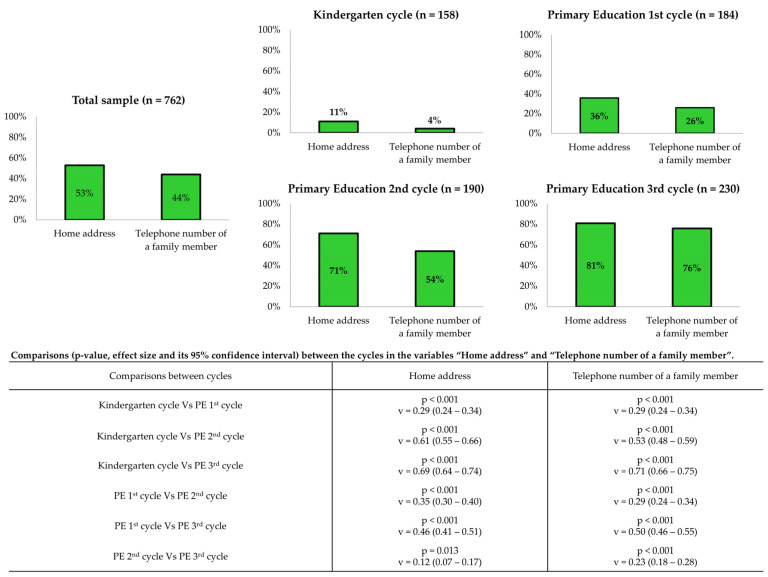
General exploratory knowledge analysis of questions asked by the dispatcher (home address and telephone number of a family member). Relative frequencies; Chi squared test (*p* < 0.05); effect Size and its 95% confidence intervals (in pairs) with Cramer’s V test: small (0.1–0.3), medium (0.3–0.5), large (≥0.5).

The results of the exploratory knowledge analysis of the key questions posed by dispatchers during emergency calls are presented in Figure 2. Of the 762 total study participants, 405 (53%) were able to provide their home address when interacting with the dispatcher, while 332 (44%) were able to provide the telephone number of a family member when requested by the dispatcher. It was observed that the percentage of students who were able to provide this information was significantly higher among students at the higher academic levels.

### 3.2. Demographic Variables of the Groups

The demographic variables of the total groups are presented in Table 3 and are segregated by cycles in the Supplementary Material. No significant differences were observed between the groups in the variables of sex, previous training in BLS, and academic years in the total sample and in each of the academic cycles.

### 3.3. Emergency Call Variables in Simulation in Total Sample

The results of emergency call variables for the total sample are presented in Table 3. A comparable proportion of participants (approximately 80% in both groups) demonstrated composure at the outset of the simulation, locating the victim’s phone, initiating an emergency call by pressing the unlock button, and dialling 112 to contact the emergency services (*p* > 0.05). Both groups demonstrated noteworthy percentages in providing the dispatcher with information regarding the victim’s circumstances.

On the other hand, the proportion of ICall-G participants who informed the dispatcher of the victim’s identity was significantly higher than that of C-G participants (94% vs. 85%; *p* < 0.001). It is also noteworthy that a significantly higher proportion of ICall-G participants (81%) activated the hands-free option when making the call compared to C-G (54%), with a *p*-value of less than 0.001.

### 3.4. Emergency Call Variables in Simulation Between Academic Cycles

**Figure 3:**
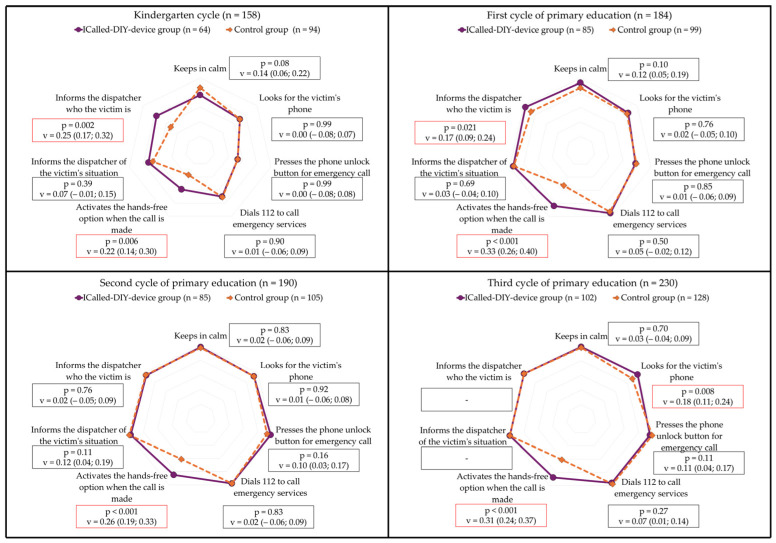
Comparison between groups in each academic cycle. Relative frequencies; Chi squared test (*p* < 0.05); effect Size and its 95% confidence intervals (in pairs) with Cramer’s V test: small (0.1–0.3), medium (0.3–0.5), large (≥0.5). Red squares indicate the presence of significant differences between groups.

The results of the emergency call variables in the academic cycles, according to groups, are presented in Figure 3 and Table A1. Upon examination of each academic cycle individually, it becomes evident that ICall-G consistently demonstrated a significantly higher proportion of participants using the hands-free option when making the call, in comparison to C-G.

### 3.5. Emergency Call Variables in Simulacion Between Academic Cycles

**Figure 4:**
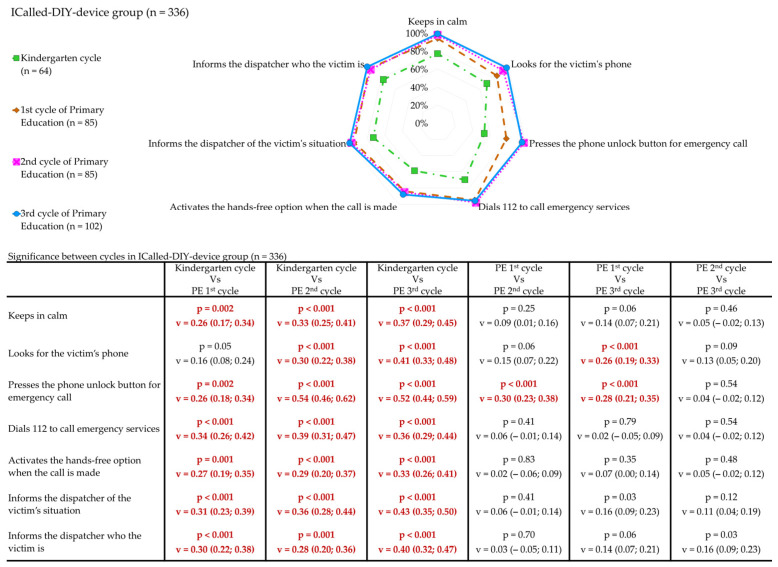
Comparison between academic cycle in the ICalled-DIY device group. Relative frequencies; Chi squared test with Bonferroni correction (*p* < 0.0083); effect Size and its 95% confidence intervals (in pairs) with Cramer’s V test: small (0.1–0.3), medium (0.3–0.5), large (≥0.5). The red font indicates the presence of significant differences between cycles.

The results of the emergency call variables in the ICall-G between academic cycles are presented in Figure 4. In this instance, no notable discrepancies were identified in any variable when comparing the 2nd-C and 3rd-C (*p* > 0.008). In the case of the 1st-C, significantly lower percentages were observed when pressing the phone unlock button to make an emergency call compared to the 2nd-C and 3rd-C. A comparison of KG-C with the remaining cycles reveals that the vast majority of variables exhibit significantly lower percentages.

## 4. Discussion

The objective of this study was to assess the efficacy of a strategy based on low-cost DIY materials for practical and adapted simulation in emergency call training (ICalled-DIY device), in comparison to a more traditional simulation strategy. The primary outcome of this study was to demonstrate that the use of the ICalled-DIY device in conjunction with a soft toy represents a more effective and efficient approach than the deployment of less adaptable and less interactive materials for children. Moreover, it has been observed that even when a strategy employing gentle and interactive teaching tools is implemented, pre-school children are not adequately prepared to learn emergency call skills as effectively as primary school children. Conversely, children aged 6–8 years have attained comparable outcomes to their older counterparts.

Almost 90% of schoolchildren were able to successfully initiate the chain of survival in an emergency situation with both training strategies. This high success rate lends further support to the existing evidence base, indicating effective learning of the skills required to activate the chain of survival [6,7]. In the specific case of the intervention with the ICalled-DIY device in conjunction with the use of a cuddly toy, this proved to be at least as effective as a conventional strategy. The ICalled-DIY device represents a cost-effective teaching tool that enables manual manipulation by schoolchildren during simulations. Furthermore, the device’s low cost and production using common school materials allows each student to create their own personalised version. The tool is perceived by schoolchildren as a toy, which, in conjunction with the use of their own soft toys, engenders a more age-appropriate and playful environment [8,9,10,11].

Furthermore, it is crucial to emphasise that the use of the ICalled-DIY device has demonstrated superior efficacy in comparison to the use of a conventional mobile phone in one particular aspect of the emergency call process: the activation of the hands-free option. Its final screen enables the user to operate the buttons once the simulated call has been initiated, at which point the hands-free option must be activated. In the case of the control group, it was not possible to access this screen when using a real smartphone, as a genuine call would be made to the emergency services. This is why, in the control group, the step of activating the hands-free option was explained to the participants but could not be demonstrated and could not be activated by them during the simulation. This limitation in the control group, which could have been circumvented by using the ICalled-DIY device, appears to have been a pivotal factor in the efficacy of learning the requisite skill to activate the hands-free mode. Although hands-free activation does not constitute a genuine impediment to the activation of the chain of survival, it constitutes a crucial step that is underscored in the recommendations of the BLS section of the ERC guidelines [13]. Therefore, an enhanced capacity to engage hands-free mode can enhance the efficiency and expediency of emergency response, facilitating more effective help to victims.

Conversely, the findings of this study indicate that the majority of kindergarten children are unaware of the home address and telephone number of a family member. These findings are consistent with those of a study conducted with children of a similar age group [14]. The results for the first cycle of primary education are similarly unpromising, aligning with the findings of other studies that have assessed these variables in this age group [7,14,15]. The lack of knowledge of this information by young people could significantly impede the effective implementation of the chain of survival. While children are capable of identifying an emergency situation, contacting emergency services, and providing accurate information, they are unable to convey the location of the emergency. The evidence provided by this study, in addition to that previously mentioned, emphasises the necessity of incorporating into training the knowledge of the addresses of the locations most frequently visited by young people (their homes and those of other family members, their schools, etc.) and the contact numbers of their relatives. The capacity to acquire this knowledge at an early age represents a significant challenge for the future of BLS training in schools. Collaborating with families outside of school hours could prove to be a highly beneficial strategy.

In relation to kindergarten schoolchildren, we also observed a notable discrepancy in their ability to learn and retain skills compared to their primary school counterparts. Nevertheless, while some limitations were observed in certain steps of the sequence, such as unlocking the phone with the emergency call button or activating the hands-free mode, approximately 70% of the participants were able to successfully locate the victim’s phone number, recognise the emergency number, and communicate it to the emergency operator in an appropriate manner. These findings are encouraging and suggest that, despite the learning of calling skills not being as effective as in more advanced stages, it is advisable to commence teaching of this content in the kindergarten cycle. In light of these findings and those of previous studies [14,15,16,17,18], it seems reasonable to posit that at least half of children between the ages of 3 and 6 would be able to activate the survival chain with adapted training. Therefore, although the assimilation of skills does not appear to be feasible at this stage, the training could prove to be lifesaving and, in turn, facilitate the acquisition of competencies at subsequent stages, making it a prudent course of action [1,5]. The most significant challenge within the content for kindergarten (KG) children appears to be the acquisition of the most frequently used addresses, adult contact numbers and other pertinent information when activating the chain of survival.

Almost 90% of participants aged 6 to 8 years demonstrated proficiency in activating the survival chain, exhibiting a performance level comparable to their older counterparts. The challenge appears to be in improving the operation of the unlock button skill and in ensure that children in this age group are aware of their parents’ home address and telephone number. It would seem that this age group is ready to assimilate this content. The results of this study, when considered alongside existing evidence, appear to suggest that these skills and content should be made mandatory at this stage [1,5].

In 2015, De Buck et al. indicated recommendations for learning objectives in BLS teaching. These included that the emergency number should be known at 6–8 years of age and reinforced at 8–12 years of age, as well as that the emergency call should start to be promoted at 6–8 years of age, be known at 8–10 years of age and reinforced at 10–12 years of age [5]. The evidence observed in this study revealed that these contents may be promoted at 3–6 years of age, should be known at 6–8 years of age, and should be reinforced at 10–12 years of age [19,20]. In contrast with this evidence, the recently enacted Spanish legislation governing school curricula does not encompass any content pertaining to BLS in the kindergarten and 6–8-year-old age groups [12].

It should be noted that this study is not without limitations. Firstly, it should be noted that the training and assessment were conducted in a simulation environment. Consequently, the results cannot be extrapolated to a real situation without taking this factor into account. Furthermore, the evaluation of the 112 call, despite the participants being able to perform all the steps that would be performed with a mobile phone, was carried out with a toy mobile phone. It should be noted that the study’s duration was insufficient to assess the long-term retention capacity of the variables under investigation. In addition, the use of soft toys as a low-cost strategy could have a positive influence on the results, although their use is due to the choice of evaluating the cheapest possible simulation strategy. Finally, the previous experience of the participants using smartphones was not assessed in this study.

## 5. Conclusions

The findings of this study indicate that the use of a practical, low-cost and adapted pedagogical instrument, designated as the DIY device, showed comparable efficacy to a conventional approach during kindergarten and primary school education. Moreover, it was demonstrated to be a more effective strategy for the implementation of hands-free mode activation during the 112 call. The findings indicate that the kindergarten stage may be an optimal period for introducing emergency call content and skills, and that children aged 6–8 are as prepared as those in later age groups for this learning.

## Figures and Tables

**Table 1:** Distribution of participants according to academic cycle and year.

Academic Cycle/Year		Total (n = 762)	ICall-G (n = 426)	C-G (n = 336)
Kindergarten cycle (KG-C)		n = 158	n = 64	n = 94
	3–4 years (4th grade-KG)	n = 33	n = 12	n = 21
	4–5 years (5th grade-KG)	n = 48	n = 19	n = 29
	5–6 years (6th grade-KG)	n = 77	n = 33	n = 44
Primary education first cycle (1st-C)		n = 184	n = 85	n = 99
	6–7 years (1st grade PE)	n = 78	n = 38	n = 40
	7–8 years (2nd grade PE)	n = 106	n = 47	n = 59
Primary education second cycle (2nd-C)		n = 190	n = 85	n = 105
	8–9 years (3rd grade PE)	n = 96	n = 53	n = 43
	9–10 years (4th grade PE)	n = 94	n = 32	n = 62
Primary education third cycle (3rd-C)		n = 230	n = 102	n = 128
	10–11 years (5th grade PE)	n = 96	n = 41	n = 55
	11–12 years (6th grade PE)	n = 134	n = 61	n = 73

ICall-G: ICalled-DIY device group (experimental); C-G: control group; KG: kindergarten; PE: primary education.

**Table 2:** Step-by-step intervention content for the theoretical and practical training sessions.

Study Phase	Duration (mins)	Content	Methodology
1. Theoretical introduction	5	Recognition of an emergency situation, steps to take (checking for consciousness, breathing) and calling emergency services.	Lecture by the nurse, with support from the teacher. Students encouraged to ask questions.
2. Instructor demonstration	5	Practical demonstration of emergency recognition and how to call 112.	Nurse models the call using a real phone (C-G) or ICalled-DIY device (ICall-G). Show how to place phone on hands-free option.
3. Student simulation and feedback	10	Students practice calling emergency services in pairs, using either a real phone (C-G) or the ICalled-DIY device (ICall-G).	Participants act out a scenario with a real phone and a training manikin (C-G) or with an ICalled-DIY device and their soft toy (ICall-G). In this scenario, they had to (1) unlock the phone screen; (2) dial 112 on the dialling screen; and (3) activate the speaker mode on the call control screen (only possible in the ICall-G). During the scenario, they received immediate instructor feedback with emphasis on (a) clarity in stating the emergency; (b) providing the correct location; (c) following instructions without hanging up.

ICall-G: ICalled-DIY device group (experimental); C-G: control group.

**Table 3:** Results of total sample (n = 762).

		ICalled-DIY Device Group (n = 336)		Control Group (n = 426)		*p* Value	Effect Size (95% CI)
		N		(%)			
Sex						*p* = 0.16	v = 0.05 (0.02; 0.09)
	Male	168	(50%)	235	(55%)		
	Female	168	(50%)	191	(45%)		
Previous training in BLS		103	(31%)	107	(25%)	*p* = 0.09	v = 0.06 (0.03; 0.10)
Keeps calm		313	(93%)	396	(93%)	*p* = 0.92	v = 0.04 (0.00; 0.08)
Looks for the victim’s phone		295	(88%)	359	(84%)	*p* = 0.17	v = 0.05 (0.01; 0.09)
Presses the phone unlock button for emergency call		281	(84%)	353	(83%)	*p* = 0.78	v = 0.01 (−0.03; 0.05)
Dials 112 to call emergency services		307	(91%)	388	(91%)	*p* = 0.89	v = 0.01 (−0.03; 0.04)
Activates the hands-free option when the call is made		271	(81%)	229	(54%)	*p* < 0.001	v = 0.28 (0.25; 0.32)
Informs the dispatcher of the victim’s situation		313	(93%)	389	(91%)	*p* = 0.35	v = 0.03 (0.00; 0.07)
Informs the dispatcher of who the victim is		314	(94%)	364	(85%)	*p* < 0.001	v = 0.13 (0.09; 0.16)

N: Absolute frequencies; (%): relative frequencies; BLS: Basic Life Support; Chi squared test (*p* < 0.05); effect size and its 95% confidence intervals (in pairs) with Cramer’s V test: small (0.1–0.3), medium (0.3–0.5), large (≥0.5).

## Data Availability

The data presented in this study are available on request from the corresponding author due to privacy and legal reasons.

## Supplementary Materials

The following supporting information can be downloaded at: https://www.mdpi.com/article/10.3390/children12030282/s1, Table S1: Evaluation checklist.

## Abbreviations

The following abbreviations are used in this manuscript:

OHCA	Out-of-Hospital Cardiac Arrest
BLS	Basic Life Support
ERC	European Resuscitation Council
CPR	Cardiopulmonary resuscitation
DIY	Do-it-yourself
KG-C	Kindergarten cycle
1st-C	Primary education first cycle
2nd-C	Primary education second cycle
3rd-C	Primary education third cycle
C-G	Control group
QCPR	Quality of cardiopulmonary resuscitation
ICall-G	ICalled-DIY Device group
IBM	International Business Machine Corporation
SPSS	Statistical Package for the Social Sciences
NY	New York
ES	Effect Size
KG	Kindergarten

## Appendix A

**Table A1:** Results of each academic cycle.

Kindergarten Cycle (n = 158)		ICalled-DIY Device Group (n = 64)		Control Group (n = 94)		*p* Value
		N	(%)	N	(%)	
Sex						*p* = 0.10
	Male	31	(48%)	58	(62%)	
	Female	33	(52%)	36	(38%)	
Previous training in BLS		4	(6%)	1	(1%)	*p* = 0.07
Keeps calm		49	(77%)	82	(87%)	*p* = 0.08
Looks for the victim’s phone		45	(70%)	66	(70%)	*p* = 0.99
Presses the phone unlock button for emergency call		34	(53%)	50	(53%)	*p* = 0.99
Dials 112 to call emergency services		45	(70%)	67	(71%)	*p* = 0.90
Activates the hands-free option when the call is made		38	(59%)	35	(37%)	*p* = 0.006
Informs the dispatcher of the victim’s situation		47	(73%)	63	(67%)	*p* = 0.39
Informs the dispatcher of who the victim is		49	(77%)	49	(52%)	*p* = 0.002
**Primary Education First Cycle** **(n = 184)**		**ICalled-DIY Device Group** **(n = 85)**		**Control Group** **(n = 99)**		***p*** **Value**
		**N**	**(%)**	**N**	**(%)**	
Sex						*p* = 0.86
	Male	50	(59%)	57	(58%)	
	Female	35	(41%)	42	(42%)	
Previous training in BLS		28	(33%)	29	(29%)	*p* = 0.59
Keeps calm		80	(94%)	86	(87%)	*p* = 0.10
Looks for the victim’s phone		71	(84%)	81	(82%)	*p* = 0.76
Presses the phone unlock button for emergency call		66	(78%)	78	(79%)	*p* = 0.85
Dials 112 to call emergency services		81	(95%)	92	(93%)	*p* = 0.50
Activates the hands-free option when the call is made		71	(84%)	52	(53%)	*p* < 0.001
Informs the dispatcher of the victim’s situation		81	(95%)	93	(94%)	*p* = 0.69
Informs the dispatcher of who the victim is		82	(97%)	86	(87%)	*p* = 0.021
**Primary Education Second Cycle** **(n = 190)**		**ICalled-DIY Device Group** **(n = 85)**		**Control Group** **(n = 105)**		***p*** **Value**
		**N**	**(%)**	**N**	**(%)**	
Sex						*p* = 0.45
	Male	39	(46%)	54	(51%)	
	Female	46	(54%)	51	(49%)	
Previous training in BLS		33	(39%)	28	(27%)	*p* = 0.07
Keeps calm		83	(98%)	102	(97%)	*p* = 0.83
Looks for the victim’s phone		79	(93%)	98	(93%)	*p* = 0.92
Presses the phone unlock button for emergency call		83	(98%)	98	(93%)	*p* = 0.16
Dials 112 to call emergency services		83	(98%)	103	(98%)	*p* = 0.83
Activates the hands-free option when the call is made		72	(85%)	64	(61%)	*p* < 0.001
Informs the dispatcher of the victim’s situation		83	(98%)	105	(100%)	*p* = 0.11
Informs the dispatcher of who the victim is		81	(95%)	101	(96%)	*p* = 0.76
**Primary Education Third Cycle** **(n = 230)**		**ICalled-DIY Device Group** **(n = 102)**		**Control Group** **(n = 128)**		***p*** **Value**
		**N**	**(%)**	**N**	**(%)**	
Sex						*p* = 0.50
	Male	48	(47%)	66	(52%)	
	Female	54	(53%)	62	(48%)	
Previous training in BLS		38	(37%)	49	(38%)	*p* = 0.87
Keeps calm		101	(99%)	126	(98%)	*p* = 0.70
Looks for the victim’s phone		100	(98%)	114	(89%)	*p* = 0.008
Presses the phone unlock button for emergency call		98	(96%)	127	(99%)	*p* = 0.11
Dials 112 to call emergency services		98	(96%)	126	(98%)	*p* = 0.27
Activates the hands-free option when the call is made		90	(88%)	78	(61%)	*p* < 0.001
Informs the dispatcher of the victim’s situation		102	(100%)	128	(100%)	-
Informs the dispatcher of who the victim is		102	(100%)	128	(100%)	-

N: Absolute frequencies; (%): relative frequencies; BLS: Basic Life Support; Chi squared test (*p* < 0.05); effect size and its 95% confidence intervals are shown in Figure 3.
