# Supplementary material for: ICalled-DIY Device for Hands-On and Low-Cost Adapted Emergency Call Learning: A Simulation Study
Source: Children (Basel). 2025 Feb 26;12(3):282. doi: 10.3390/children12030282 (PMC11941231; doi:10.3390/children12030282)
Supplement: Supplementary file 1 [file children-12-00282-s001.zip › children-3489988-supplementary.pdf]

Participant code:			
Evaluation date:			
"You are playing in your room, and you hear a noise in the living room. As you approach the living room, you see the relative who was with you at home lying on the floor. What should you do?"			
Does the participant remain calm?	Yes		No
Is the participant looking for the teacher's smartphone?	Yes		No
Does the participant press the phone unlock button for emergency call?	Yes		No
Does the participant dial 112 to call emergency services?	Yes	He/she dials another number	No
Does the participant activate the hands-free option when the call is made?	Yes		No
Does the participant know how to explain to the dispatcher what is going on?	Yes		No
Does the participant know how to explain to the dispatcher to whom this is happening?	Yes		No
Does the participant know how to explain his/her home address to the dispatcher?	Yes		No
Does the participant know how to tell the dispatcher the telephone number of an adult family member?	Yes		No
